# Distal radius fractures - evidence based approach

**DOI:** 10.1186/1753-6561-9-S3-A35

**Published:** 2015-05-19

**Authors:** Daniel Herren

**Affiliations:** 1 Department of Hand Surgery, Schulthess Klinik, Zurich, 8008. Switzerland

## 

Distal radius fractures account for the most commonly treated fracture of the upper extremity and are especially common in the elderly population. Despite new treatment strategies many questions remain regarding the optimal treatment regime. Controversies include the treatment modalities and the expected outcome as well as economical considerations. It is the goal of this presentation to discuss five common questions and statements around distal radius fractures in the light of the published evidence based literature.

## Statement 1: Most distal radius fractures are nowadays best treated with volar plating

Several studies, including those analyzing patient population older than 65 years old, suggest that operative treatment with volar plating does not provide better clinical results compared to other treatment modalities, especially conservative treatment with repositioning and plaster immobilization. There is evidence that in the earlier stage of fracture healing, there is a faster return to activities of daily living provided by open reduction and internal fixation (ORIF) but at 6 months follow-up the results of different treatment modalities are similar. More complex fractures seem to benefit from ORIF in both radiographical and clinical results. In addition, radiographical results are superior in ORIF but with no significant influence on the clinical results.

## Statement 2: In distal radius fractures concomitant ulna styloid fractures need not to be fixed

There are several studies suggesting that distal radio-ulnar joint function is not affected by whether the ulna styloid is healed or not healed after the treatment of distal radius fractures. As long as the anatomy of the DRUJ is well reconstructed, even in ulna styloid base fractures DRUJ instability seems to be rare. However, there is no standardized description of the assessment of DRUJ stability as well intra-as postoperatively and therefore no recommendation can be given in this regard. In addition it is unknown how much TFCC scarring contributes to DRUJ re-stabilization of distal radio-ulnar joints. On the other hand, ulna styloid re-fixation, regardless of the technique used, has a relatively high potential of complications, especially DRUJ stiffness and disturbance including painful neuroma formation of the sensory branch of the ulnar nerve.

## Statement 3: There is a clear tendency of publication bias in the literature of distal radius fractures and a lack of randomized controlled studies

Together with the development of new treatment concepts and especially new plating systems, the amount of articles concerning the outcome of distal radius fracture treatment has nearly exploded. According to the analysis of Sando et al 2013, there is a clear tendency for publication bias in distal radius fracture literature. Significantly more reports about positive outcome are published (70%) compared to only 5% of publications with negative outcomes. The authors state that a more standardized approach to measure results and to judge possible publication bias should be implemented. Especially in pathologies as frequent as distal radius fractures, more randomized controlled studies should be available, in order to enhance the level of evidence to enlighten certain pending questions.

## Statement 4: Intra-articular steps of > 2 mm in intra-articular distal radius fractures have a significant higher likelihood of cartilage degeneration in the long-term

Since the publication of Knirk and Jupiter 1986 about the outcome of distal radius fractures, the ‘magic’ > 2mm rule of inacceptable intra-articular step in the treatment of distal radius fractures have been burnt into the brains of hand surgeons. A later analysis of the study by the authors themselves advocated significant methodological flaws and they had to admit that the conclusion drawn at the time do not withstand the principles of evidence based medicine. Other publications warn of the correlation between radiographical appearance and functionality in the long-term. However there are no studies with sufficient power to answer the question on the amount of tolerable intra-articular step and the long-term association.

Different reasons might be responsible for this lack of scientific data. It might be speculated that with new treatment concepts the level of fracture fixation improved significantly in the last 30 years and less fractures with articular steps are accepted. In addition, it seems unethical to randomized patients in different groups with more or less articular steps in order to answer this question.

## Statement 5: Volar plating is associated with a high number of complications

The increased use of volar plates in the treatment of distal radius fractures has led to more reports on its complications. The number of problems, which lead to further treatment, is in most series between 5-10% for volar plating. Two major types of problems might be identified: hardware associated complications and surgical approach associated problems. Basically both can be summarized as technical issues and in most cases avoidable. Several studies therefore advocate, that with increasing surgeon’s experience, fewer complications are to be expected. However, besides malunion, only non-operative treated distal radius fractures seem to have less complications than all other different type of operative procedures for this problem. Nevertheless for a fair comparison, there is a clear lack of randomized controlled studies with enough statistical power.

